# Correction to: Epigenetic targeting of the ACE2 and NRP1 viral receptors limits SARS‑CoV‑2 infectivity

**DOI:** 10.1186/s13148-021-01195-2

**Published:** 2021-11-22

**Authors:** Maria Laura Saiz, Marta L. DeDiego, Darío López-García, Viviana Corte-Iglesias, Aroa Baragaño-Raneros, Ivan Astola, Victor Asensi, Carlos López-Larrea, Beatriz Suarez-Alvarez

**Affiliations:** 1 Translational Immunology Laboratory, Health Research Institute of Asturias (ISPA), Oviedo, Spain; 2 Department of Molecular and Cellular Biology, Centro Nacional de Biotecnología (CNB-CSIC), Madrid, Spain; 3 Intensive Care Department, Hospital Universitario Central de Asturias, Oviedo, Spain; 4 Translational Microbiology Research Group, Instituto de Investigación Sanitaria del Principado de Asturias (ISPA), Oviedo, Spain; 5 Infectious Diseases Unit, Translational Research in Infectious Diseases Group, Hospital Universitario Central de Asturias, Instituto de Investigación Sanitaria del Principado de Asturias (ISPA), OviedoOviedo, Spain; 6 Department of Immunology, Hospital Universitario Central De Asturias, Oviedo, Spain

## Correction to: Clin Epigenet (2021) 13:187 10.1186/s13148-021-01168-5

Following publication of the original article [1], the authors identified errors in Fig. 7 and in the co-author name. Panels A and B of Fig. 7 were the same as panels C and D. At the last name of Marta L. De Diego the space between “De” and “Diego” must be removed. The correct name is “Marta L. DeDiego”. These have been corrected with this erratum.

The corrected Fig. 7 is given below:

**Fig. 7:**
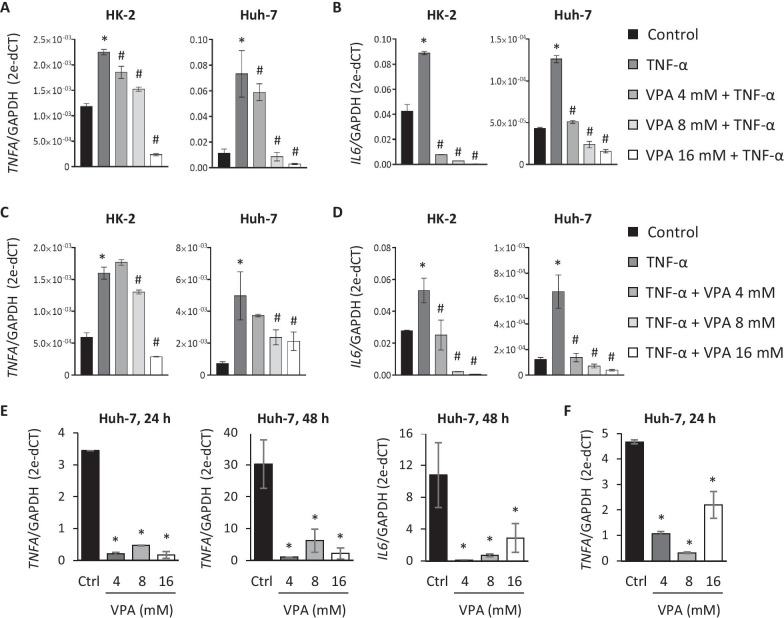
Treatment with VPA inhibits the inflammatory response triggered by TNF-α induction and SARS-CoV-2 infection. **a**,** b** HK-2 and Huh-7 cells were treated with VPA (4, 8, and 16 mM) for 24 h and (10 ng/ml) was added in the final 3 h. **c, d** HK-2 and Huh-7 cells were cultured with TNF-α (10 ng/ml) for 3 h and without removing the culture medium, and VPA (4, 8, and 16 mM) was added for an additional 24 h. TNF-α and IL-6 expression was quantified by RT-qPCR and represented as n-fold induction over the levels of mock-treated cells. **e** Huh-7 cells were treated with VPA (4, 8, and 16 mM) for 24 h before SARS-CoV-2 infection (MOI 0.5), and TNF-α and IL-6 expression was evaluated by RT-qPCR at 24 and/or 48 hpi and represented as the n-fold induction over the levels of mock-infected cells. **f** Huh-7 cells were infected with SARS-CoV-2 and 1 hpi, cells were left untreated (control) or treated with 4, 8, and 16 mM of VPA for 24 h. TNF-α expression was analyzed by RT-qPCR and represented as the n-fold induction over the levels of mock-infected cells. All samples were normalized relative to GAPDH expression using the 2^−ΔCT^ method. Data are represented as the mean ± SD of at least three independent experiments. *p < 0.05

The original article has been corrected.

## References

[1] Saiz ML (2021). Epigenetic targeting of the ACE2 and NRP1 viral receptors limits SARS-CoV-2 infectivity. Clin Epigenet.

